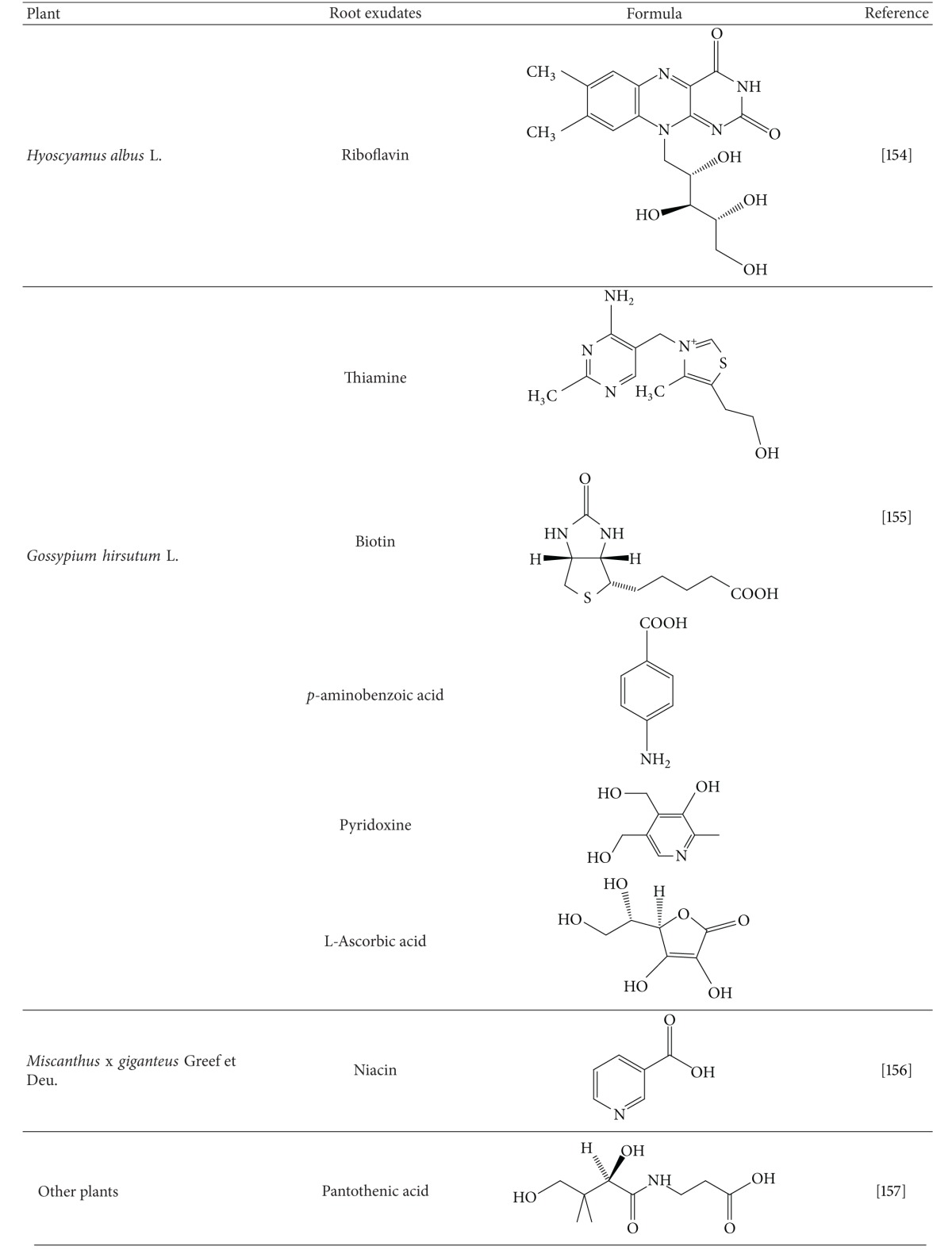# Aliphatic, Cyclic, and Aromatic Organic Acids, Vitamins, and Carbohydrates in Soil: A Review

**DOI:** 10.1155/2013/524239

**Published:** 2013-11-10

**Authors:** Valerie Vranova, Klement Rejsek, Pavel Formanek

**Affiliations:** Department of Geology and Soil Science, Mendel University in Brno, Zemedelska 3, 613 00 Brno, Czech Republic

## Abstract

Organic acids, vitamins, and carbohydrates represent important organic compounds in soil. Aliphatic, cyclic, and aromatic organic acids play important roles in rhizosphere ecology, pedogenesis, food-web interactions, and decontamination of sites polluted by heavy metals and organic pollutants. Carbohydrates in soils can be used to estimate changes of soil organic matter due to management practices, whereas vitamins may play an important role in soil biological and biochemical processes. The aim of this work is to review current knowledge on aliphatic, cyclic, and aromatic organic acids, vitamins, and carbohydrates in soil and to identify directions for future research. Assessments of organic acids (aliphatic, cyclic, and aromatic) and carbohydrates, including their behaviour, have been reported in many works. However, knowledge on the occurrence and behaviour of D-enantiomers of organic acids, which may be abundant in soil, is currently lacking. Also, identification of the impact and mechanisms of environmental factors, such as soil water content, on carbohydrate status within soil organic matter remains to be determined. Finally, the occurrence of vitamins in soil and their role in biological and biochemical soil processes represent an important direction for future research.

## 1. Introduction

Organic acids, vitamins, and carbohydrates play an important role in soil. Organic acids (aliphatic, cyclic, and aromatic) play key roles in rhizosphere ecology, pedogenesis, nutrient acquisition, allelochemical interactions, availability and detoxification of aluminium and pollutants, regulation of soil pH, enzymatic activities, and in food-web interactions [1–9].

Carbohydrates represent dominant compounds of plant root exudates. They play an important role in the establishment and functioning of mycorrhizal symbioses and the stabilisation of heavy metals in soil [10–12]. Determination of soil carbohydrates is mostly related to the evaluation of the effect of land use change on soil organic matter status, particularly in terms of microbial transformation [13–15].

While there is little knowledge on occurrence of vitamins in soil, vitamins are known to play a number of important roles in plants including resistance to pathogens, plant-microbe symbioses, microbial growth stimulation, and stimulation of organic pollutant degradation [16–19].

## 2. Organic Acids in Soil

### 2.1. Aliphatic Organic Acids

A wide range of organic acids has been found in soil. These include aliphatic acids such as acetic, citric, isocitric, fumaric, tartaric, oxalic, formic, lactic, malic, malonic, butyric, succinic, *trans*-aconitic, propionic, adipic and glycolic acids, and cyclic and aromatic acids such as benzoic, phenylacetic, shikimic, phthalic, ferulic, syringic, *p*-coumaric, vanillic, *p*-hydroxybenzoic, *m*-hydroxybenzoic, benzoic, caffeic, protocatechuic, gallic, gentisic, sinapic, rosmarinic, and transcinnamic acids [3, 20–33].

Knowledge of the behaviour of aliphatic organic acids in soil in terms of nutrient acquisition by plants, microbial degradation and adsorption, their role in pedogenesis and in Al detoxification, extraction, and analysis was reviewed by Jones [1], Jones et al. [2], and Van Hees et al. [34]. Separation of low molecular weight organic acid-metal complexes by HPLC was reviewed by Collins [35]. Organic acids were reported to form 4% of dissolved organic carbon (DOC) and up to 27% of acidity in mor layers of coniferous forests [36, 37]. Individual aliphatic organic acids occur in soils from different ecosystems in concentrations up to 6000 *μ*M and within individual ecosystems, and the broadest spectrum of these acids was found in forest soils (Table 1).

Concentrations of aliphatic organic acids commonly decrease with soil depth, except in the case of some ecosystems such as those containing podzolized soils, where organic acids (e.g., formic acid) reportedly increased in concentration with depth [38]. Of the individual organic acids, fumaric acid was present in higher concentrations in mineral horizons of alkaline soils [45], while citric acid was reported in concentrations of between 20 and 1000 *μ*M in upper soil layers [21, 34, 38, 46]. Citric acid played the most important role in terms of buffering capacity [24].

Organic acids are involved in the formation of complexes of Al and Fe. The amount of complexed Al and Fe declines with soil depth [47]. Different organic acids play a role in the formation of complexes of Al and Fe within soil profiles. For example, citric acid has been reported as the most important complexing agent in O and E horizons, whereas oxalic acid is reported to play the most significant role in horizon B [47]. Citric, oxalic, and malic acids are thought to be particularly important in rhizosphere ecology and pedogenesis [2, 5, 6].

The primary production rate of organic acids in different types of soils was predicted to be within the range of between <1 and 1250 nmol/g soil/d [6]. Acetic and formic acids increased in concentration with decomposition of wood chips during a mycoremediation process [48]. Low molecular weight organic acids are thought to be responsible for minimizing crop damage by the root-knot nematode *Meloidogyne incognita* (Kofoid and White (Chitwood)) [49]. Production of gluconic acid by rhizosphere soil bacteria presents an efficient strategy to avoid protozoan grazing. Gluconic acid was shown to cause encystment or death of protozoa [9]. Succinic acid decreased the growth and conidial germination of *Fusarium oxysporum *f. sp. *niveum *[50], while propionic, acetic, lactic, malic, and citric acids were all demonstrated to have significant antibacterial effects [51].

Organic acids were found to increase the activity of acid phosphomonoesterase in soil at low concentrations (<1 *μ*mol/g), whereas higher concentrations (>5 *μ*mol/g) of citric, oxalic, malic, and tartaric acid inhibited this activity [52]. Organic acids also act as adsorbents of acid phosphomonoesterase [4] from minerals and colloids (desorption by up to ca. 60%). This indicates the changes in behaviour of acid phosphomonoesterase in the rhizosphere, where organic acids are released from plant roots, compared to bulk soil. Organic acids in soil are produced by plant root exudation and by activity of soil microorganisms. Phosphate-solubilizing bacteria (*Bacillus*, *Rhodococcus*, *Arthrobacter*, *Serratia*, *Chryseobacterium*, *Delftia*, *Gordonia,* and *Phyllobacterium*) which increase P-uptake by plants were reported to produce aliphatic organic acids such as citric, gluconic, lactic, propionic, and succinic acids [53].

 Average respiration rates of organic acids (oxalate, citrate) were reported to be around 209 nmol/g soil/d, and respiration of organic acids increased with soil depth [6, 39]. Van Hees et al. [21] and Ström et al. [54] reported rapid degradation of citric, malic, and oxalic acid in most soils. In some cases, organic acid degradation may be inhibited by complexation with Ca (oxalate in calcareous soils); degradation of individual organic acids may also differ between rhizosphere and bulk soil [39, 54]. Forest soils differ in their abilities to anaerobically consume organic acids such as oxalate. The addition of electron donors (acetate, glucose, vanillate, or hydrogen) or acceptors (nitrate or sulphate) did not affect anaerobic consumption of oxalate, whereas CO_2_ or bicarbonate totally repressed it [55].

There is a paucity of literature on organic acid enantiomers, but what does exist points to the need for urgent study. Liao et al. [25] identified D-tartaric acid in concentrations up to 6 *μ*g/g in the rhizosphere of *Lactuca sativa* L., which, along with L-citric acid, formed the dominant organic acid. A recent review has highlighted the potential importance of future research in this area [56].

#### 2.1.1. Role of Aliphatic Organic Acids in Soil Decontamination

Organic acids play an important role in the phytoremediation of polluted soils and in the availability of heavy metals and organic compounds. Mobilisation of polycyclic aromatic hydrocarbons (PAHs) such as pyrene or phenanthrene by organic acids (citric, oxalic, tartaric, lactic, or acetic) is dependent on the type of organic acid, pH, and soil organic matter content [8, 57]. For example, citric acid has been reported to be efficient in pyrene and phenanthrene extraction [8, 57]. Lower extractability of PAHs was found in soils of higher organic matter content, while adsorption of pyrene in the presence of organic acids decreased with increasing pH. Citric and malic acids inhibited adsorption of chemotherapeutics in soil. Soil pH, surface properties, and competitive adsorption of other cations affected this process [58].

Introduction of *Bacillus thuringiensis* (B_*t*_) to soil, as a result of rapid planting of B_*t*_-transformed crops, may cause hazards for soil ecosystems; thus, the factors affecting its mobility need to be determined. Organic acids (citric, oxalic, and acetic) are one of the factors affecting mobility of B_*t*_ toxin in soil. Fu et al. [59] reported decreased adsorption of B_*t*_ toxin by minerals such as kaolinite, goethite, and silicon dioxide due to low concentrations of organic acids, whereas high concentrations of these acids promoted adsorption of the toxin. Increasing concentrations of oxalate and citrate inhibited adsorption of B_*t*_ toxin by montmorillonite.

Organic acids such as citric and tartaric acids were found to reduce Cr(VI) to Cr(III) in the soil [60] and to affect mobility of heavy metals due to their desorption, complexation, and precipitation. Dissolution of minerals in fly ash from smelters allowed conversion of heavy metals to their mobile forms [61–64]. Cu phytoextraction by *Nicotiana tabacum* L. was enhanced by citrate, whereas Pb phytoextraction was not stimulated by aliphatic organic acids, probably due to the rate of degradation of organic acids in soil, which is reported to be high for metals of low mobility and bioavailability [65].

Generally, citric acid is the most effective in terms of desorption of different heavy metals, followed by malic > acetic > tartaric > oxalic acid (Cu, Hg, Pb, Cd, Zn, and ^137^Cs) [66–73]. Schwab et al. [72] found citric acid to be the most efficient in desorption of Zn and Cd in sandy loam, but it had little impact on Pb movement. Desorption of heavy metals in soil by organic acids depends on the concentration and degradability of the organic acids, pH, and concentration of competing cations such as Ca^2+^ [61, 62, 74]. Effective mobilisation of Zn in soil due to formation of citrate-Zn complexes was reported by Lombnæs et al. [62]. Citric acid rapidly degrades, even in heavy metal-polluted soils, with 20% degradation between 1 and 4 days being reported by Wen et al. [74]. Fast degradation of organic acids in soil leads to low migration [6, 54]. On the other hand, complexation of organic acids with Al slightly decreases their degradation [5]. Metal complexes of organic acids differ in their microbial degradability, with higher degradation for citrate-metal complexes compared to oxalate-metal complexes [68].

Huang et al. [75] reported a stimulating effect of low molecular weight organic acids for Cd and Pb adsorption by goethite and montmorillonite, but only at low concentrations. At higher concentrations of these acids, decreased heavy metal adsorption was recorded. While citric and tartaric acids enhanced desorption of Cu in soil, oxalic acid was effective in desorption of Cu and Cd [61]. The mechanism of desorption was explained as competition in complexation, adsorption, and precipitation. Gao et al. [76] reported desorption of Cd and Cu by citric and tartaric acids, especially at higher concentrations. Low concentrations of these acids inhibited desorption.

Organic acids appeared to be efficient in the release of ^137^Cs from contaminated soils, efficiency being in the order citric > tartaric > oxalic > succinic > acetic acid [73]. Desorption occurs in two phases: fast and slow. The fast stage of desorption corresponds with the interaction of organic acids with the surface of clay minerals, whereas the slow stage (occurring over a much longer period) is attributed to inter- and intraparticle diffusion. Debela et al. [64] reported the release of Pb from pyromorphite [Pb_5_(PO_4_)_3_Cl] by citric, malic, acetic, and oxalic acids. Interestingly, low concentrations of organic acids may increase adsorption of heavy metals in soil [77].

### 2.2. Cyclic and Aromatic Organic Acids in Soil

Cyclic and aromatic organic acids play a range of roles in soils, including allelopathic interactions, inhibition of microbial growth, and weathering of minerals [78, 79]. Some aromatic acids in soil solution may also be used to distinguish between vegetation types in forests [40]. Asao et al. [3] reported that benzoic, *m*- and *p*-hydroxybenzoic, vanillic, and adipic acids inhibited plant growth. Of these, benzoic acid was the strongest inhibitor. Ferulic acid is released from plant roots and from decomposition of soil organic matter and may be involved in allelopathic interactions. Caspersen et al. [80] reported the presence of bacteria in commercial hydroponic *Lactuca sativa* L. culture which were able to ameliorate the toxic effects of ferulic acid.

Aromatic acids (salicylic and phthalic) are adsorbed by soils of different charges, and the adsorption of these acids differs significantly according to the soil tested. Adsorption of aromatic and aliphatic acids decreased the zeta potential of soils and oxides [81, 82]. Adsorption of salicylate in soil appeared to be significantly lower compared to citrate (Freundlich constant for adsorption K_*F*_ 0.499 versus 0.107) [69]. Adsorption of gallic acid was not influenced by soil depth or land use [26]. Gallic acid decreased the amount of total inorganic nitrogen extractable from soil by KCl and increased solubility of Ca and Mn through formation of metal-gallic acid complexes and redox reactions. However, gallic acid did not affect extraction of total soluble-N.

Inderjit and Bhowmik [27] reported sorption of benzoic acid in soil which increased with its concentration, with a nonlinear adsorption isotherm. The authors reported sorption to be sufficiently strong to protect plants from phytotoxic effects of this compound and to be pH-dependent. Benzoic acid is reversibly adsorbed to soil particles by van der Waal or hydrogen bonding and can be released to soil solution due to decreasing strength of the soil solution or presence of competing ions [83]. Evans Jr. [84] reported decreasing degradation of phthalic acid with depth in forest soil. Shikimic acid was detected in mor layer extracts in concentrations of 12 *μ*M [37]. Shikimic acid (even in a large quantity) did not affect decomposition of citrate, malate, and oxalate in agricultural soils [85] and had a low effect on sorption of these acids. Oburger et al. [85] reported the half-life for shikimic acid in different soils to be within a range from 0.6 to 8.6 h. Caffeic acid inhibited growth of *Frankia* isolates [79], while gentisic, *o*-hydroxyphenylacetic, and vanillic acid were less inhibitory.

#### 2.2.1. Role of Cyclic and Aromatic Organic Acids in Availability of Heavy Metals

Cyclic and aromatic organic acids affect availability of heavy metals in soils. Whereas salicylic acid decreased availability of Pb, the presence of phthalic or salicylic acid increased the capacity of exchangeable Al. In some of the tested soils, salicylic acid decreased the capacity due to its lower adsorption and its formation of soluble Al-salicylate complexes [69, 82]. The ability of aromatic acids to mobilize Al is lower compared to a range of aliphatic organic acids (citric, oxalic, malonic, malic, and tartaric) but was higher than in the cases of lactic or maleic acid [86, 87]. Mobilisation of Al by salicylic acid was decreased by increasing pH.

Some aromatic acids, such as gallic acid, are efficient in extraction of heavy metals (Cd, Cu, Zn, and Ni) [70]. Weathering of minerals (e.g., labradorite ((Ca,Na)(Si,Al)_4_O_8_) or microcline (KAlSi_3_O_8_)) by formation of Al-organic complexes by salicylic acid was reported by Huang and Keller [78]. Salicylic and phthalic acid release Cu from chalcopyrite (CuFeS_2_) and release Ca and P from apatite (Ca_5_(PO_4_)_2.82_(FeClOH)_1.54_) [88]. Salicylic and phthalic acid are less efficient in release of yttrium from phosphate minerals (apatite, monazite) than citrate; phthalate efficiency is comparable to oxalate [89].

## 3. Carbohydrates in Soil

Glucose, galactosamine, fructose, rhamnose, arabinose, fucose, glucosamine, galactose, xylose, mannose, ribose, mannosamine, muramic, galacturonic, and glucuronic acids have all been identified in soil [15, 28, 90–96]. Tian et al. [60] reported ca. 30% of DOC in arable soils was formed by carbohydrates, representing 4–7% of total organic carbon [97]. The annual flux of carbohydrates infiltrating mineral soil of *Picea abies* (L.) H. Karst. stands was assessed by Guggenberger et al. [98] to be ca. 70 kg/ha/y. Sugars as well as phenolic compounds are chemoattractants of rhizobacteria [99, 100]. Carbohydrates alleviate negative effects of wood ash on enchytraeid growth and abundance, possibly by correcting an imbalance in the bacteria: fungi ratio, which is increased by addition of wood ash [101]. Glucuronic, galacturonic, and alginic acids (main constituents of bacterial exopolymeric substances) play a role in stabilisation of heavy metals such as Cr (VI) in soil under acidic or slightly alkaline conditions [12]. The ratio of carbohydrate C/polyphenol C in soil hydrolysates is used as an indicator of soil organic matter quality [102], and the ratio of total carbohydrates/K_2_SO_4_ extractable total N appears to be a good predictor of N mineralisation and microbial biomass N [103].

Adsorption of carbohydrates, such as glucose or fructose, on alumina interfaces is characterised by an adsorption isotherm of a typical L-type, and an adsorption mechanism based on dipolar interaction has been suggested [90]. The adsorption was pH dependent and was affected by anions (Cl^−^, SO_4_^2−^, and PO_4_^3−^) and cations; fructose appeared to be better adsorbed than glucose. Pentoses (arabinose and xylose) are not synthesised by microorganisms and are constituents of plant biomass. On the other hand, galactose, mannose, rhamnose, and fucose are of microbial origin [14, 104] and up to ca. 16 mg/g soil organic carbon from a range of different soils was ascribed to microbial sugars [105]. According to Oades [106], the ratio of galactose plus mannose/arabinose plus xylose is low (<0.5) for plant-derived sugars and high (>2) for microbial sugars.

Amino sugars represent major constituents of microbial cell walls and hydrolysable soil organic matter. Free amino sugars represent a small part of the dissolved organic C and N pools [107]. Muramic acid, glucosamine, mannosamine, and galactosamine may be used as an indicator of microbial origin of soil organic matter [108, 109]. Glaser et al. [110] reported that total amino sugar and muramic acid in soil microbial biomass varied between 1 and 27 mg/kg soil, while microbial biomass made a negligible contribution to total amino sugar concentration in soil. Glucosamine and galactosamine were found in the highest concentrations in different horizons of forest and prairie soils (up to 5200 mg/kg soil) [108, 109].

Carbohydrates from soil microbial biomass were reported by Joergensen et al. [111] to account for 17% of total carbohydrate C, and the content of microbial biomass carbohydrates correlated well with microbial biomass C [112]. Carbohydrates are extracted from soil using cold or hot water, 0.5 M K_2_SO_4_, 0.25 M H_2_SO_4_, 1 M HCl, 0.5 M NaOH, or 4 M trifluoroacetic acid [13, 105, 111, 113, 114]. Adesodun et al. [115] and Ball et al. [13] reported extraction of the lowest carbohydrate fraction (3%) using cold water, 10% by hot water, 12% by 1 M HCl, and 75% by 0.5 M NaOH.

### 3.1. The Role of Carbohydrates in Aggregation

Mineral-organic associations represent a large amount of carbon in terrestrial ecosystems; these associations have a high abundance of microbially derived carbohydrates [116]. Plant carbohydrates depend on texture type, being higher for loamy sand than silt loam [117]. Carbohydrates play an important role in the formation of stable aggregates [118]. Fungi increase aggregate stability, due to a supply of extracellular polysaccharides [119]. On the other hand, Adesodun et al. [115] reported that aggregate stability correlated very poorly with carbohydrates fractions. Aggregate stability seems to better correlate with carbohydrates in hot water or dilute acid extracts, indicating suitability of these types of extracts to indicate changes in soil due to land use change [120].

Microaggregates (20–53 *μ*m) had a higher ratio of mannose plus galactose/arabinose plus xylose than other aggregate fraction of larger sizes up to >212 *μ*m (macro- and meso-), indicating the importance of microbial processes. Solomon et al. [14] reported an increase of neutral sugars and uronic acids in particle size fractions, in the order silt < coarse sand < fine sand < clay. Soil organic matter in nano-size structures isolated from a clay fraction accumulated carbohydrates between groups of other compounds (N-heterocyclics, peptides, and alkyl aromatics) [121]. Puget et al. [122] found increasing carbohydrates with aggregate size, clay, and silt fractions within stable aggregates.

### 3.2. Carbohydrates in Different Soil Types and Depths

Soil type has an impact upon sugar synthesis by microorganisms, reflecting microbial biodiversity and varied ecophysiology between soils. Derrien et al. [123] quantified sugar synthesis in soil from ^13^C labelled substrates using compound-specific isotope ratio mass spectrometry. The authors reported that the quality of added substrate (mono- and polysaccharide or amino acid) had little effect upon sugar production in soil.

The concentration of carbohydrates generally decreases with soil depth [105, 124]. Carbohydrate content decreased from litter to soil organic matter and aggregates with incorporation of soil [125]. Carbohydrates can accumulate in horizons with strongly humified organic matter probably due to the toxic effect of adsorption to some oxides or hydroxide minerals, especially those with aluminium content. Minerals such as ferrihydrite and aluminium hydroxide reduced carbohydrate decomposition by 15–50% [124].

Osono et al. [126] reported a higher content of soluble carbohydrates in bleached litter colonised by *Clitocybe* sp. than in nonbleached litter. Carbohydrates are amongst the more rapidly degraded compounds of plant litter, resulting in organic matter being more enriched in lignin-derived compounds [127]. The ratio of selected hexoses to pentoses in needles was 1–15 times lower compared to decomposing litter [128].

Rumpel et al. [129] evaluated the effect of soil type on carbohydrate content and found that carbohydrate content was generally higher in Cambisol than Podzol. Sugars were enriched in mineral-bound fractions of organic matter, often with microbial monosaccharides. On the other hand, bulk soil was characterised by higher contributions of plant-derived sugars. The type of extractant has an effect on the proportion of carbohydrates in total organic C within a profile. Water-soluble carbohydrates are generally not proportional to the total organic carbon content in soil [130]. The ratio of hydrolysable carbohydrate C/total organic C increased with soil depth, with an increasing importance of cellulosic polysaccharides in the B horizon. In hot water extracts, the ratio was similar throughout the whole profile [131, 132]. Sugars (other than cellulosic) were maintained at a relatively constant level within the soil profile (12–15% of organic carbon).

Generally, glucose was found in the highest concentrations in the upper humus layer [131]. The importance of microbially derived sugars increased with soil depth [105]. The ratio of mannose plus galactose/xylose plus arabinose increased from the litter layer to the H horizon, indicating the increasing importance of microbially derived sugars. The type of extractant used has an effect on the ratio of galactose plus mannose/xylose plus arabinose. Hot-water extraction was 1–1.6 compared to a NaOH extraction, with the ratio 0.4–0.7 indicating a higher microbial contribution in hot-water extracts [13]. Verchot et al. [118] reported decreased concentrations of carbohydrates in soil with depth; arabinose and mannose were the most abundant sugars within aggregate fractions (micro-, meso-, macro-, and bulk soil). Amino sugars were also found to decrease downward in the profiles [133].

A high level of water (in Bg horizon) negatively affects the proportion of amino sugars within the total organic carbon. Enhanced drying of soil decreased the contribution of plant and microbial sugars to soil organic matter in the O and A horizons even though the sugar content of the original plant material increased with drying [105]. However, the concentration of mannitol and trehalose (stress-induced fungal metabolites) increased at low soil moisture [134].

### 3.3. The Effect of Land Use on Soil Carbohydrates

The concentration of soluble sugars in soils from different ecosystems changes over the course of the vegetative season [113, 134] and is affected by the type of plant coverage, soil properties, and microbial activity. The concentration of pentoses during a growing season corresponded with litterfall, ground grass cutting in forest sites, drying of grass in grasslands, and harvest in agroecosystems [135].

Management of ecosystems may affect carbohydrate quantity, quality, and distribution within soils [13, 14, 136, 137]. Generally, management of soil has no effect on the occurrence of dominant carbohydrates in soil hydrolysates (Table 2). Carbohydrate content in soils will increase in a number of situations, including integrated crop-livestock systems, cultivated fields compared to tropical woodlands, establishment of pasture on acid savanna soils, arable compared to fallow sites, manuring, application of organic wastes such as poultry manure or composts in saline soils, larvae (*Trpula paludosa*), addition of *Aspergillus niger* with *Beta vulgaris* L. wastes, inoculation with *Bacillus cereus*, mixing of mineral soil with the litter layer, forests compared to pastures or cropland, elevated CO_2_, reduction of fungicides, mycorrhizal inoculation, and the addition of *Beta vulgaris* L. or rock phosphate [14, 60, 98, 103, 113, 120, 138–148]. The type of management of arable land influences distribution of soil carbohydrates, being more uniform within depth in ploughed compared to drilled soils [13].

Manure application, crop rotation, and avoiding tillage for 6 years all increased amino sugar content in soil [120, 138]. Amino sugar content was at its highest on plots with continuous *Zea mays* L. monoculture (up to 1317 mg/kg) compared to a *Zea mays* L.—*Glycine max* (L.) Merr. rotation field [158]. Carbohydrates (especially glucose and xylose) are dominant components of dung [120, 138] and are thought to contribute significantly to carbon stock and aggregate stability in manured soils, replacing the existing pool. A maximum of 60% of dung-derived C was found as carbohydrates after 56 days incubation. Management of land has effects on the utilisation of dominant compounds in water-soluble root exudates. For example, nontilled plots had higher microbial utilisation of carboxylic acids and lower utilisation of amino acids and carbohydrates compared to conventionally tilled or rotatory-tilled soils [159]. Stevenson et al. [160] reported higher utilisation of carbohydrates and amino acids and lower utilisation of carboxylic acids in soils of pasture relative to forest soils.

In terms of other treatments, UV-B radiation reduced extractability of carbohydrates from leaf litter of *Quercus robur *L., thus changing litter carbon source availability for soil microorganisms [161]. The ratio of rhamnose plus fucose/xylose plus arabinose increased on the forest floor and in the coarse fraction of topsoil after forest dieback [162]. The ratio of mannose plus galactose/xylose plus arabinose was higher in C-depleted than fertilised plots with the highest value in fine particles [163].

Change in land use (e.g., pasture to arable land) also causes a new equilibrium for soil carbohydrates, established after 14 and 56 years [139]. Carbohydrates occurred in higher concentration in macroaggregates than microaggregates, and the ratio of distribution of carbohydrates between macroaggregates and microaggregates did not change over 110 years. No effect of arable soil fertilisation (organic versus mineral) on the occurrence of sugars (rhamnose, xylose, glucose, mannose, arabinose, and galactose) in soil hydrolysates was reported by Lima et al. [28]. Eleven years after liming of *Picea abies* (L.) H. Karst. stands, no significant changes in the carbohydrate fraction were found by Rosenberg et al. [164].

Soil carbohydrate levels have also been reported to decrease during boreal forest succession, root exclusion, grazing of semiarid shrubland, conversion of pasture to cropland, and during conversion of forests on sandy spodosols to *Zea mays* L. cropping [15, 97, 136, 137, 165]. Amino sugar content decreases with afforestation, cultivation of plots related to grassland, and during clear-cutting of forest related to cultivated sites [93, 97]. The application of fungicides may significantly change concentrations of some sugars in soil (e.g., mannose). Earthworms reduced the concentration of xylose and glucose, suggesting accelerated turnover of plant material in the soil [136].

## 4. Vitamins in Soil

Knowledge of the quantity of vitamins in soils of different ecosystems is poor. Sulochana [155] found pyridoxine, thiamine, *p*-aminobenzoic acid, and traces of biotin in soil. Barrera-Bassols et al. [166] suggested that *Quercus robur *L. litter could contain high vitamin content, but experimental proof is currently lacking. Soil algae produce vitamin signals (lumichrome and riboflavin) that act as agonists within bacterial communities through quorum sensing [167]. Vitamins are also known to act as attractants to *Caenorhabditis elegans*.

Vitamins may be important in the decontamination of polluted soils and were reported to stimulate PAHs degradation [19] and attenuation of alkanes in oil-polluted desert soil [16, 19]. Vitamins added to soil increased the rate of degradation of 2,4,6-trinitrotoluene (TNT) [168]. The addition of vitamins B_1_ + B_6_ + B_12_ enhanced the growth of fungi in the presence of phenol [169], while the addition of a vitamin solution containing biotin, folic acid, riboflavin, niacin, and thioctic acid increased phenolic degradation by between 7 and 16% [170]. Minor adsorption of vitamin B_12_ on kaolinite clay and sand, with no detectable adsorption to alumina, was reported by Hashsham and Freedman [171].

Vitamins (riboflavin, vitamin B_12_, niacin, thiamine, ascorbic and pantothenic acid, *p*-aminobenzoic acid, biotin, *β*-carotene, pyridoxine, and tocopherol) [154, 172–174] enter soil from different sources including root exudation (Table 3), plant biomass, and bacterial production [154, 172, 174–177]. For example, the distribution of vitamin E (*α*-, *β*-, and *γ*-tocopherol) in *Picea abies* (L.) H. Karst. was reported by Franzen et al. [178]. While *α*-tocopherol was found in all organs, *β*- and *γ*-tocopherol were restricted to seedlings and seeds. Phosphate-solubilising bacteria, azotobacters, and rhizobia are significant producers of vitamins [172, 174, 179, 180]. Hodson et al. [180] isolated the soil bacterium *Mesorhizobium loti*, whose genome sequence is known to support growth of the vitamin B_12_ auxotroph *Lobomonas rostrata*. Application of some insecticides may inhibit microbial production of vitamins in soil by bacteria such as *Azospirillum brasilense *[181].

## 5. Conclusions

Aliphatic, cyclic, and aromatic organic acids play an important role in soil and rhizosphere ecology, as well as in decontamination of polluted sites. Despite much work on the occurrence and behaviour of organic acids in soil, current knowledge is mostly restricted to their L-enantiomers. In future research, determination of the occurrence and role of D-enantiomers of organic acids in soil and rhizodeposition should become a significant focus, particularly relating to their potential in allelopathic interactions, decontamination of polluted sites, and in terms of their roles in plants suitable for phytoremediation purposes. Carbohydrates represent an abundant group within soil organic matter, serving as an indicator of the quality of soil organic matter and of land use changes. Despite the existence of a broad literature on soil carbohydrates and their fractionation within soils across many ecosystems, there still remains a paucity of research on the effects of environmental factors, especially altered soil water content, on qualitative and quantitative changes in soil carbohydrates. Vitamins play an important role in biochemical soil processes and decontamination of polluted sites. More research is needed on their occurrence and behaviour in soil.

## Figures and Tables

**Table 1:** Dominant organic acids in soil of different ecosystems.

Management	Dominant organic acids	Ratio aliphatic/cyclic plus aromatic acids	Concentrations	Sampling	Increase/decrease with depth	References
*Pinus sylvestris *L*., Quercus* *robur* L., *Picea abies* (L.) H. Karst., *Betula* *pendula* Roth., *Fagus* sylvatica L., and *Abies alba* Mill.	Citric, acetic, formic, oxalic, malic, butyric, propionic, malonic, lactic, tartaric, succinic, shikimic, and propionic acid	4–157	Up to 5820 *μ*M*	Whole profile	Mostly decrease, sometimes increase with depth	[6, 20–22, 36–41]

*Lupinus polyphyllus* Lindl., *Agropyron repens* L., *Juncus effusus* L., *Juncus inflexus* L., and* Juncus* *articulatus* L.	Citric, acetic, formic, lactic, and oxalic acid	4–10	Up to 1 *μ*mol/g soil	A-horizon	—	[23, 42, 43]

Contaminated soils (industrial, agricultural)	Oxalic acid	—	Up to 3 *μ*mol/g soil	—	—	[44]

*Dissolved organic matter was extracted from the fresh A horizon soil samples using double-deionized water with a solid/volume ratio of 1 : 2.

**Table 2:** Carbohydrates in soil collected from different ecosystems.

Management	Type of extraction	Dominant carbohydrates	Concentrations	Sampling (horizon or depth)	References
Rotation of vegetables, legumes, and *Triticum aestivum* L.	Solution	Glucose, glucuronic, and galacturonic acid	0.115 *μ*g/g	Ah	[91]

Arable land (different managements)	Hot water and NaOH extract	Arabinose, xylose, mannose, galactose, glucose, and rhamnose	Up to 358 *μ*g/g	0–10, 0–60 cm	[13]

Arable land (different rotation, crops, organic and mineral fertilization, biotic treatments, etc.)	Hydrolysate	Xylose, arabinose, galactose, glucose, and mannose,	Up to 4000 *μ*g/g	0–30 cm	[28, 118, 136, 138, 149, 150]

Forests (*Salix phylicifolia* L., *Alnus incana *L. Moench., *Betula* *pubescens *L. and *Picea abies* (L.) H. Karst., *Quercus robur* L., and *Fagus sylvatica* L.)	Hydrolysate	Xylose, glucose, galactose, arabinose, and mannose	Up to 253 · 10^3^ *μ*g/g organic carbon	Different horizons	[15, 151, 152]

Grasslands	Hydrolysate	Glucose, galactose, arabinose, mannose, and xylose	More than 700 *μ*g/g	0–75 cm	[149, 150, 153]

Savannah	Hydrolysate	Glucose, mannose	Up to 2000 *μ*g/g	0–10 cm	[149]

Shrublands	Hydrolysate	Galactose, glucose, arabinose, and xylose	Up to 2400 *μ*g/g	0–5 cm	[137]

Prairie	Hydrolysate	Arabinose, galactose, xylose, and glucose	Up to 4000 *μ*g/g	—	[150]

Four soil types (vegetation not specified)	Hydrolysate	Glucose, galactose, mannose, arabinose, and xylose	Up to 2000 *μ*g/g	0–20 cm	[92]

**Table 3:** Vitamins in plant root exudates.